# Hyperglycemia—A Driver of Cutaneous Severity in Dermatomyositis: A Narrative Review

**DOI:** 10.3390/jcm15020734

**Published:** 2026-01-16

**Authors:** Rachel Dombrower, Alyssa McKenzie, Olga Gomeniouk, Savannah Kidd, Shannon Saed, Sophia Saed, Erin Onken, Juwairiah Mohammad

**Affiliations:** 1 School of Medicine, St. George’s University, University Centre Grenada, West Indies 11739, Grenada; allyrosemckenzie@gmail.com; 2 Rush Medical College, Rush University, Chicago, IL 60612, USA; 3 School of Medicine, Southern Illinois University, Springfield, IL 62702, USA; 4 CUNY School of Medicine, City College of New York, New York, NY 10031, USA; 5 Sophie Davis School of Biomedical Education, City College of New York, New York, NY 10031, USA; 6 Edward Via College of Osteopathic Medicine, Blacksburg, VA 24060, USA; 7 Jackson Memorial Hospital, 1611 NW 12th Ave., Miami, FL 33136, USA

**Keywords:** dermatomyositis, cutaneous dermatomyositis, hyperglycemia, metabolic dysfunction, autoimmunity, AGEs, microvasculopathy, inflammation, wound healing

## Abstract

Dermatomyositis (DM) is an idiopathic inflammatory myopathy (IIM) characterized by distinctive chronic cutaneous manifestations. Although immune-mediated and microvascular mechanisms are well established, the role of metabolic dysfunction, particularly hyperglycemia, is underexplored in dermatological conditions. This review synthesizes mechanistic, clinical, and translational evidence to explore the relationship between dysglycemia and cutaneous disease severity in DM. Hyperglycemia is associated with oxidative stress, advanced glycation end-product formation, endothelial injury, and proinflammatory cytokine signaling. These processes may plausibly amplify DM-associated vasculopathy, impair wound healing, and worsen cutaneous inflammation. Limited DM-specific studies demonstrate increased insulin resistance and a higher prevalence of diabetes compared with healthy controls. Meanwhile, case reports suggest that poor glycemic control can exacerbate cutaneous disease. Evidence from other inflammatory dermatoses supports a biologically plausible role for dysglycemia in increasing flare frequency, infection risk, and delayed tissue repair. Dietary patterns characterized by high glycemic index and coexisting metabolic syndrome may further intensify systemic and cutaneous inflammation. Collectively, these findings suggest hyperglycemia as a biologically plausible contributor to cutaneous disease severity in DM that warrants further investigation. These observations highlight the need for future studies to evaluate whether metabolic screening, dietary patterns, and interdisciplinary care influence cutaneous disease activity and wound healing in DM. Prospective clinical investigation is needed to determine whether targeted glycemic optimization is associated with changes in cutaneous and systemic outcomes in DM.

## 1. Introduction

Dermatomyositis (DM) is a rare idiopathic immune-mediated inflammatory disease (IMID) characterized by proximal muscle weakness and distinctive cutaneous manifestations including heliotrope rash, Gottron’s papules, and photosensitive eruptions [1,2,3]. Cutaneous involvement is a defining feature of DM and may precede, accompany, or persist independently of muscle disease, contributing substantially to disease burden and quality-of-life impairment.

DM demonstrates a bimodal age distribution affecting both pediatric and adult populations, with a higher prevalence in women than in men [1,2]. Although clinical outcomes vary, many patients experience a chronic disease course with recurrent flares, and cutaneous manifestations are often persistent and refractory to standard immunosuppressive therapies [2].

DM is characterized by myositis-specific autoantibodies, type I interferon response, complement-mediated microangiopathy, and cutaneous vasculopathy [4]. Although these processes are central to muscle and skin injury, the potential role of metabolic disturbances in shaping cutaneous inflammatory activity remains underexplored in dermatology-focused literature.

The pathogenesis of DM is multifactorial, involving genetic, immunologic, and environmental influences. Metabolic factors, particularly hyperglycemia and insulin resistance, may influence disease severity through pathways that overlap with DM-related cutaneous injury [5]. Chronic hyperglycemia promotes systemic inflammation, impairs immune regulation, and can exacerbate both muscular and cutaneous manifestations of DM [5,6]. Hyperglycemia and insulin resistance are associated with oxidative stress, advanced glycation end-product (AGE) formation, endothelial dysfunction, and impaired wound healing. Despite this mechanistic overlap, dermatology-focused literature has not fully examined how glycemic status influences cutaneous DM expression.

By integrating hyperglycemia with established pathogenic mechanisms, including endothelial injury, AGE accumulation, oxidative stress, and inflammatory cytokine amplification, this review reframes metabolic dysregulation as a potentially modifiable driver of cutaneous disease activity. Accordingly, this review focuses specifically on cutaneous manifestations and the dermatologic factors influencing disease severity, rather than systemic myositis outcomes. In the context of DM, “cutaneous severity” refers to the extent and activity of inflammatory skin disease as assessed by validated clinical instruments and clinically meaningful dermatologic outcomes. In the existing literature, cutaneous disease activity is most commonly quantified using the Cutaneous Dermatomyositis Disease Area and Severity Index (CDASI), which evaluates erythema, scale, and damage across affected body sites [7,8]. Additional measures used to characterize cutaneous severity include the presence and persistence of ulceration, vasculopathic lesions, poikiloderma, refractory pruritus, impaired wound healing, secondary infection, and reduced responsiveness to standard dermatologic therapies [3]. In this review, cutaneous severity encompasses both validated activity scores (e.g., CDASI) and clinically relevant manifestations reflecting inflammatory burden, tissue injury, and healing impairment.

Given the limited availability of DM-specific interventional studies, this review adopts a narrative framework integrating mechanistic, observational, and translational evidence from related immune-mediated inflammatory skin diseases.

## 2. Methods

This narrative review examined the relationship between glycemic dysregulation and cutaneous disease activity in DM. A literature search was conducted using PubMed/MEDLINE for English-language articles published between January 2000 and December 2025. Search terms included combinations of “dermatomyositis,” “cutaneous dermatomyositis,” “skin manifestations,” “hyperglycemia,” “diabetes,” “insulin resistance,” “glycemic control,” “advanced glycation end products,” and “metabolic syndrome,” with manual screening of reference lists to identify additional relevant studies. Eligible publications included original research articles, observational studies, clinical trials, mechanistic studies, and relevant reviews addressing DM, immune-mediated inflammatory skin disease, metabolic dysregulation, or glycemic effects on cutaneous inflammation and wound healing. Non-English articles, conference abstracts without full text, case reports lacking mechanistic relevance, and studies unrelated to cutaneous or inflammatory pathways were excluded. Owing to limited DM-specific data, evidence from related immune-mediated inflammatory dermatoses and metabolic disease literature was incorporated to support biologic plausibility, with study selection and interpretation performed narratively and guided by relevance to cutaneous inflammation, vasculopathy, and wound repair.

## 3. Pathophysiological Background

The pathophysiology in DM is multifactorial and involves autoantibody generation, T-cell-driven inflammation, cytokine imbalance, and vascular injury. T-lymphocytes coordinate the inflammatory response and promote B-cell activation, leading to the production of disease-specific autoantibodies. These mechanisms directly contribute to both muscle damage and the distinct dermatologic features of the disease.

Several autoantibodies are implicated in DM, including anti-Mi-2, anti-TIF1γ, anti-MDA5, anti-NXP2, and anti-SAE [9]. These myositis-specific autoantibodies define distinct clinical phenotypes that influence organ and skin involvement, with variable cutaneous severity and associated features including photosensitivity, vasculopathy, and systemic involvement [9,10]. Beyond defining phenotypes, these autoantibodies also shape prognosis and inform individualized management approaches. For example, anti-TIF1γ is associated with aggressive cutaneous disease and malignancy risk, whereas anti-MDA5 correlates with ulcerative skin lesions and rapidly progressive interstitial lung disease [9]. These autoantibodies provide prognostic insight and affect dermatological manifestations, often preceding aggressive muscle disease [9].

Downstream immune signaling and vascular injury mediate cytokine release and tissue damage associated with DM. In skin and muscle, elevated levels of type I interferons, interleukin (IL)-6, IL-18, and tumor necrosis factor-alpha (TNF-α) sustain inflammatory cascades and promote keratinocyte injury, contributing to persistent cutaneous inflammation and skin barrier disruption. A hallmark pathological feature of DM is microvascular injury initiated by autoantibody-triggered deposition of the membrane attack complex (C5b-9) on endothelial cells, resulting in capillary damage, ischemia, perifascicular atrophy, and cutaneous vasculopathy [11,12]. These vascular injuries manifest as violaceous erythema, poikiloderma, ulceration, and the characteristic heliotrope rash and Gottron’s papules. Ongoing cutaneous vasculopathy further impairs wound healing and re-epithelialization, increasing susceptibility to ulcerative and necrotic disease [11,12].

Immune dysregulation and microvascular injury define the clinical manifestations of DM. DM presents with symmetric proximal muscle weakness and distinctive dermatological features, including heliotrope rash, Gottron’s papules, V-sign, and shawl sign [2,13]. Less common but clinically important features include calcinosis cutis, particularly in younger patients, and “mechanic’s hands,” characterized by hyperkeratosis, fissuring, and scaling along the lateral fingers [9,10]. These immune, vascular, and inflammatory mechanisms create a pathogenic substrate upon which metabolic dysfunction and hyperglycemia may further amplify cutaneous inflammation and exacerbate skin involvement in DM.

Type I interferon signaling is a central pathogenic driver in DM and contributes to cutaneous inflammation and microvascular injury through sustained expression of interferon-stimulated genes (ISGs). Although direct evidence in DM is lacking, hyperglycemia-related metabolic stress may plausibly intersect with this pathway. Oxidative stress, mitochondrial dysfunction, and AGE–RAGE signaling induced by hyperglycemia can activate inflammatory transcriptional programs that overlap with interferon-regulated signaling. In keratinocytes and endothelial cells, such metabolic stress may lower the threshold for ISG expression and potentiate interferon-mediated inflammation and vascular injury. These interactions remain hypothesis-generating and warrant future investigation in DM.

Throughout this review are distinct metabolic terms used with specific meanings. “Hyperglycemia” refers to elevated circulating glucose levels, typically defined by fasting plasma glucose ≥ 126 mg/dL or HbA1c ≥ 6.5%, consistent with diagnostic criteria for diabetes mellitus. “Dysglycemia” is used as an umbrella term encompassing abnormal glucose homeostasis, including impaired fasting glucose, impaired glucose tolerance, and overt diabetes. “Insulin resistance” denotes reduced cellular responsiveness to insulin and may be present with or without frank hyperglycemia. The term “diabetes mellitus” is reserved for established clinical diagnoses based on standardized glycemic thresholds. Where possible, distinctions between these entities are maintained; yet, some cited studies report metabolic abnormalities using overlapping or surrogate measures, which is acknowledged in the interpretation of findings.

## 4. Hyperglycemia and Cutaneous Manifestations

**Figure 1:**
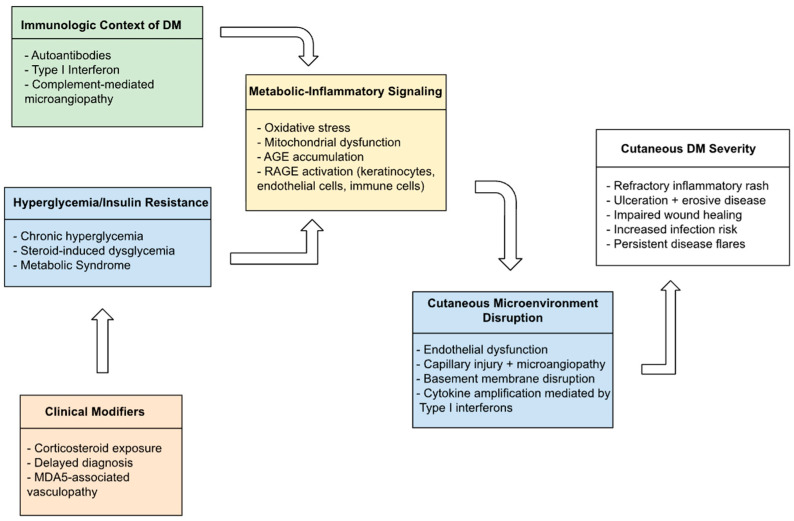
Integrated pathophysiologic model demonstrating the effects of dysglycemia on cutaneous DM.

Hyperglycemia may modify cutaneous disease activity in DM by altering inflammatory signaling, endothelial integrity, and tissue repair. Although direct interventional studies evaluating the impact of hyperglycemia on cutaneous outcomes in DM are limited, evidence from mechanistic studies, observational DM cohorts, and related inflammatory dermatoses supports biologic plausibility. The mechanisms by which dysglycemia amplifies cutaneous disease severity in DM are summarized in Figure 1.

At the cellular level, hyperglycemia activates several pro-inflammatory pathways through oxidative stress and NF-κB activation, which increase the production of pro-inflammatory cytokines and chemokines [14]. Hyperglycemia also induces mitochondrial damage, thereby promoting reactive oxygen species generation, and accelerates AGE formation [14]. AGEs bind to the receptor for advanced glycation end products (RAGE) on keratinocytes, endothelial cells, and immune cells, sustaining a cytokine-rich inflammatory environment and promoting endothelial dysfunction [15]. This pro-inflammatory environment can worsen skin involvement in DM, manifesting as more severe rashes, delayed wound healing, and heightened disease flares.

Clinical observations suggest that metabolic dysregulation is present early in the course of DM and influences cutaneous disease activity independent of immunosuppressive treatment exposure [5]. Subclinical insulin resistance and impaired glucose homeostasis amplify inflammatory signaling in autoimmune disease and are associated with increased susceptibility to cutaneous inflammation, delayed wound healing, and disease flares, particularly in individuals with underlying metabolic risk factors [16]. Poor glycemic control may further compound endothelial dysfunction and barrier impairment, thereby exacerbating vasculopathic features and chronic cutaneous involvement. Although there is a paucity of DM-specific interventional data, converging clinical and mechanistic evidence supports dysglycemia as a driver of amplified cutaneous inflammation, impaired repair processes, and worsened healing in immune-mediated skin disease.

Sustained dysglycemia exacerbates inflammatory and microvascular abnormalities in DM, contributing to worsening cutaneous disease expression and impaired disease control [5]. Poor glucose regulation promotes endothelial dysfunction, disrupts epidermal barrier integrity, and delays tissue repair [15]. Collectively, existing clinical evidence indicates that dysglycemia influences cutaneous inflammation, barrier dysfunction, and impaired healing outcomes across immune-mediated skin disease [16].

These findings align with observations in other inflammatory dermatoses in which poor glycemic control is associated with more severe outcomes. For instance, in patients with psoriasis, coexisting diabetes or prediabetes with elevated HbA1c (≥7%) are at increased risk for disease exacerbation [17]. In patients with hidradenitis suppurativa, ingestion of high-glycemic index (GI) foods can trigger rapid glucose spikes, leading to increased insulin and IGF-1, downregulation of FOXO1, thus promoting follicular hyperproliferation, and intensified inflammation in high-friction areas [18]. Similarly, HbA1c > 7.5% was associated with a 1.4-fold increased risk of infection among patients with cellulitis, suggesting that hyperglycemia impairs wound healing and may provide a nutrient source for bacterial proliferation [19]. Together, these studies indicate that metabolic dysregulation amplifies cutaneous inflammation, delays healing, and exacerbates disease severity through mechanisms highly relevant to DM. The principal pathophysiologic domains through which glycemic dysregulation may influence cutaneous disease severity in DM are summarized in Table 1.

Hyperglycemia and diabetes mellitus occur across idiopathic inflammatory myopathies (IIMs), including polymyositis and overlap syndromes, reflecting shared contributors such as systemic inflammation, glucocorticoid exposure, reduced physical activity, and baseline metabolic risk [20]. Importantly, diabetes prevalence increases with advancing age independent of IIM or DM status, and adult DM cohorts frequently include individuals at higher background metabolic risk [21]. Hyperglycemia observed in DM populations may partly reflect age-related susceptibility rather than disease-specific mechanisms alone. As cutaneous disease is a defining feature unique to DM, its vasculopathic and interferon-driven skin pathology may confer heightened susceptibility to the pro-inflammatory and endothelial effects of dysglycemia, suggesting a disease-modifying role for hyperglycemia despite largely associative evidence.

Importantly, metabolic considerations are also relevant in juvenile dermatomyositis (JDM). Although JDM differs from adult-onset disease in clinical course and treatment exposure, pediatric patients may similarly experience dysglycemia due to prolonged systemic corticosteroid use, chronic inflammation, and reduced physical activity during critical periods of growth. While direct evidence linking hyperglycemia to cutaneous disease severity in JDM is lacking, these metabolic disturbances could plausibly influence wound healing, vasculopathic skin lesions, and long-term morbidity, underscoring the need for further investigation across age groups.

## 5. Dietary and Lifestyle Contributions

Dietary patterns and lifestyle behaviors represent modifiable contributors to systemic inflammation and skin disease activity in DM. Evidence from other immune-mediated inflammatory diseases indicates that dietary interventions can alter disease activity, despite limited DM-specific data.

Consumption of high-GI foods acutely elevates postprandial glucose and triglyceride levels, activating inflammatory pathways. Biomarkers such as glycoprotein acetylation and IL-6 have been shown to increase following consumption of high–glycemic index foods, primarily in studies conducted among healthy individuals or populations with metabolic syndrome rather than patients with IIMs [22]. These findings provide mechanistic insight into post-prandial inflammatory responses but may not directly reflect disease-specific effects in DM. In contrast, meta-analyses of diets comprising foods with a low-GI index show inconsistent effects on other inflammatory markers, including C-reactive protein (CRP) and TNF-α [23]. This heterogeneity suggests that inflammatory responses to dietary glycemic load vary according to metabolic status, disease phenotype, or gut microbiome composition of individuals [24,25,26]. Although evidence specific to DM is lacking, patterns associated with diets comprising foods with a high GI could exacerbate systemic inflammation and worsen cutaneous activity, particularly in patients with insulin resistance.

No formal dietary guidelines for DM are available. In other IMIDs such as rheumatoid arthritis, inflammatory bowel disease, and multiple sclerosis, diets high in saturated fat, refined sugars, and ultra-processed foods are associated with increased systemic inflammation, whereas diets rich in fruits, vegetables, fiber, and omega-3 fatty acids are linked to improved health outcomes [27,28,29,30]. A standardized tool that may be used is the Dietary Inflammatory Index (DII) which quantifies the relationship between dietary intake and systemic inflammation by assigning evidence-based scores to nutrients and foods based on their documented associations with inflammatory biomarkers, including CRP, IL-6, and TNF-α [31,32,33]. Higher DII scores correspond to more proinflammatory dietary patterns linked to systemic inflammation and metabolic dysfunction, although the DII has not been validated as a tool that can be utilized in DM [27,34]. Nevertheless, DII-based frameworks offer a structured means of examining how diet-related inflammatory burden may influence cutaneous disease activity in DM.

Metabolic syndrome and insulin resistance are highly prevalent in DM and are linked to greater disease severity and progression. Reported prevalence of metabolic syndrome ranges from 25% to 42% among adults with DM, exceeding that observed in healthy controls [35,36,37]. In one cohort study, the rate of diabetes among patients with DM was nearly 18%, compared with the 1% present among controls [35]. Diets with higher inflammatory potential correlate with hypertension, abdominal obesity, hypertriglyceridemia, and insulin resistance [38]. DM is also associated with dysregulated lipid metabolism and impaired fatty acid oxidation, which correspond with higher disease activity [39]. Therefore, these findings suggest that dietary modification targeting metabolic syndrome and insulin resistance may improve DM outcomes, with confirmation in interventional studies still required.

Among dietary patterns studied in immune-mediated inflammatory diseases, the Mediterranean diet most consistently reduces systemic inflammation and improves metabolic parameters in patients with IMIDs. The Mediterranean diet emphasizes fruits, vegetables, whole grains, legumes, nuts, seeds, fish, and olive oil and limits red meat, refined carbohydrates, and added sugars. Furthermore, it is rich in low-GI foods and associated with improved glycemic control and reduced postprandial glucose excursions [40,41,42]. Adherence to this diet is linked to reduced CRP and IL-6 levels, improved body composition, lower triglycerides, and enhanced insulin sensitivity in patients with chronic inflammatory and metabolic conditions [43,44,45]. Given the overlap between metabolic dysfunction and DM pathophysiology, the Mediterranean diet may be particularly well suited for dietary interventions in this population. Importantly, dietary considerations in DM must balance metabolic goals with the increased protein requirements associated with inflammatory myopathy, and low–glycemic index frameworks should be interpreted within this broader nutritional context rather than as standalone dietary guidance.

Diets characterized by a high-GI warrant particular attention, as postprandial hyperglycemia may exacerbate inflammatory burden. The correlations between insulin resistance, metabolic syndrome, and worsened outcomes in DM support dietary approaches that improve glycemic control as adjuncts to medical therapy. Future research should evaluate the effects of low-GI index and Mediterranean-style diets on cutaneous and systemic manifestations of DM to identify modifiable lifestyle factors associated with improved patient outcomes. The major metabolic and lifestyle contributors relevant to cutaneous inflammation and disease severity in DM are summarized in Table 2.

## 6. Connections with Other Autoimmune Conditions

DM is associated with an increased risk of comorbid autoimmune conditions that reflect broader patterns of immune dysregulation [2]. While precise understanding of autoimmunity pathogenesis is incompletely understood, connections between immune dysregulation and environmental or microbial triggers exist [26]. Clinical observations demonstrate a correlation between DM and type 1 diabetes mellitus, an insulin-dependent autoimmune condition [16]. However, the temporal relationship between these conditions remains uncertain, and it is unclear whether shared genetic susceptibility or environmental triggers contribute to their co-occurrence.

Viral infections are potential triggers of DM and type 1 diabetes mellitus. Viruses including Coxsackie B, toxoplasma, human T-cell leukemia virus-1, echovirus, influenza, and human immunodeficiency virus are linked to inflammatory myopathies, while viral-induced beta-cell destruction has been implicated in autoimmune pathogenesis of type 1 diabetes mellitus. Additionally, steroid treatment for DM can precipitate hyperglycemia or steroid-induced diabetes, necessitating careful glucose monitoring during therapy [3,46,47]. These findings are especially relevant to dermatological management because fluctuations in glucose levels can worsen cutaneous disease activity.

Genetic predisposition also contributes to the development of both DM and type 1 diabetes mellitus. Shared variants in genes such as CFTR, PLB1, CD6, tyrosine hydroxylase, DNAH2, and PRF1 have been implicated in both conditions, particularly in the juvenile-onset forms [48]. In reported cases of DM with coexisting type 1 diabetes mellitus, patients exhibit classic clinicopathologic features of DM, including perivascular and interstitial mononuclear cell infiltration on muscle biopsy and myopathic changes on electromyography [2,48,49]. These genetic overlaps may partly explain the more severe cutaneous manifestations and greater flare frequency in DM patients with additional autoimmune diseases.

Steroid therapy is commonly used to treat DM and alleviate cutaneous and muscular inflammation, but it frequently induces hyperglycemia, which can reveal latent diabetes mellitus and exacerbate cutaneous and systemic disease manifestations [50]. This risk is particularly relevant in patients with dermatological diseases, as elevated blood glucose levels can worsen skin lesions and complicate autoimmune disease management, thereby reinforcing a vicious cycle of metabolic and inflammatory dysregulation [50].

Beyond endocrine comorbidity, DM is closely associated with paraneoplastic autoimmune activity and ovarian, lung, pancreatic, gastric, colorectal, and breast malignancies [48,49]. The risk of cancer is greatest within the first three years of initial DM diagnosis, with adult patients exhibiting a three- to five-fold higher risk compared with the general population [51,52]. Malignancy-associated DM often presents with prominent cutaneous features, like necrotic lesions, severe vasculopathy, and ulcerative disease, which can provide dermatologists with early clues prompting oncologic evaluations [53,54]. Although hyperglycemia is not directly implicated in paraneoplastic DM, treatment-induced metabolic disturbances and systemic inflammation may indirectly amplify cutaneous involvement.

These associations highlight the close relationship between DM and coexisting autoimmune and systemic events, including diabetes and other metabolic disorders, with important prognostic implications for cutaneous disease. Dermatologists should be highly suspicious of associated autoimmunity, monitor for steroid-related hyperglycemia, and consult with endocrinologists, rheumatologists, oncologists and other interdisciplinary specialists when indicated. Awareness of these overlapping conditions can facilitate earlier detection and management of risk factors that may worsen dermatological severity.

## 7. Prognostic Considerations and Clinical Relevance

While DM is a multisystem disease, this discussion focuses on prognostic considerations related to cutaneous severity, wound healing, and overall dermatologic disease burden. The impact of hyperglycemia on internal organs is well recognized, but its effects on skin health receive less attention. As outlined in Section 3, hyperglycemia-related metabolic disturbances may impair skin barrier integrity and wound repair, increasing vulnerability to injury and infection [55,56,57,58]. In DM patients, particularly those with MDA5-associated vasculopathy or chronic ulcerative lesions, metabolic dysregulation may further impair an already compromised repair environment [59]. Therefore, regular skin monitoring and early glycemic management are essential in DM, both as preventive measures and as strategies to support effective treatment responses.

Hyperglycemia has been associated with impaired wound healing and increased infection risk in other clinical contexts. Elevated blood glucose levels impair immune function and create conditions conducive to bacterial proliferation [60]. Evidence from surgical and experimental models demonstrates that elevated glucose levels delay re-epithelialization and increase susceptibility to infection [61,62]. While these findings are not DM-specific, they provide biologic plausibility for similar vulnerabilities in DM, where vasculopathic lesions and incomplete re-epithelialization may predispose to secondary infection. Even modest elevations in glucose levels can adversely affect healing at high-risk cutaneous sites [62]. These observations highlight the need for future studies examining whether glycemic status influences wound outcomes and infection risk in cutaneous DM.

Targeted studies should investigate glycemic control as a modifiable factor of DM treatment outcomes. Given the prevalence of insulin resistance and metabolic dysfunction in DM, future studies should assess whether optimizing blood glucose levels improves disease activity, cutaneous manifestations, and muscle involvement. Such studies should also examine correlations between glycemic markers (e.g., HbA1c, fasting glucose, insulin resistance indices) and histopathologic features in DM biopsies, such as endothelial swelling, capillary dropout, thrombi, and ulceration depth or chronicity. Future studies may clarify whether metabolic interventions, including glycemic optimization, are associated with changes in cutaneous disease activity or healing outcomes in DM.

Interventional studies directly evaluating the impact of glycemic optimization on cutaneous disease activity in DM are needed. Much of the existing understanding is inferred from other inflammatory dermatoses, which may not fully capture the unique vasculopathic and reparative features of DM. Prospective studies that correlate glycemic markers with cutaneous severity, ulceration, histopathologic features, and treatment response are therefore needed. Ultimately, prospective evidence is required to determine whether metabolic assessment and glycemic management are associated with improved cutaneous outcomes in DM.

## 8. Future Considerations for Dermatologic Research and Care

**Figure 2:**
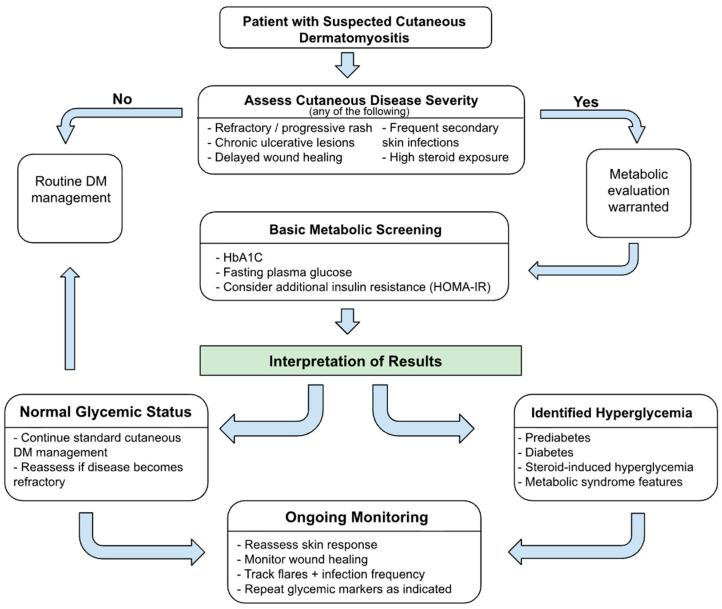
Dermatology-focused algorithm for metabolic evaluation and management in patients with cutaneous dermatomyositis.

Metabolic dysregulation represents a potentially modifiable but incompletely characterized factor that may influence the dermatologic course of DM [5,35]. Dysglycemia is a clinically relevant contributor to disease burden and should be considered in the dermatologic management of wound healing, ulceration, and refractory cutaneous inflammation. Since dermatologists are frequently the first clinicians to diagnose DM based on skin findings, early identification of metabolic risk factors through targeted glycemic assessment may represent an area for future investigation in relation to cutaneous disease activity. A conceptual dermatology-centered framework illustrating how glycemic assessment could be explored in cutaneous DM research is shown in Figure 2.

### 8.1. Screening and Evaluation

Evaluation of metabolic parameters may represent a future area of investigation in patients with active cutaneous DM, particularly those with persistent, poorly healing, or ulcerative lesions. Assessment of HbA1c, fasting glucose, and indices of insulin resistance could help clarify whether metabolic dysfunction is associated with chronic inflammation and impaired wound healing in this population. This hypothesis may be especially relevant in patients with pronounced vasculopathy, significant poikiloderma, or frequent disease flares. In patients with established diabetes or steroid-induced hyperglycemia, collaboration with endocrinology may be relevant in future multidisciplinary care models but is not currently evidence-based for DM-specific cutaneous outcomes.

### 8.2. Treatment Considerations

A mainstay for DM management includes systemic steroids, thus monitoring for steroid-induced hyperglycemia is necessary [46]. These considerations are discussed to highlight potential interactions between treatment-related metabolic effects and cutaneous disease activity rather than to propose changes to current treatment standards. Dermatologists should be aware that high-dose or prolonged systemic corticosteroid therapy may induce hyperglycemia, which could theoretically influence wound healing and cutaneous inflammation. Steroid-sparing regimens and adjunctive immunomodulators are discussed here as conceptual strategies that may reduce treatment-related metabolic burden, although their impact on glycemia-driven cutaneous outcomes in DM has not been directly studied. Glycemic monitoring and metabolic management are presented as areas for future research and should not be interpreted as substitutes for disease-directed immunomodulatory therapy. During corticosteroid tapering, metabolic risk may fluctuate, but the relationship between dose reduction and glycemic normalization in DM has not been systematically studied. There is currently insufficient evidence to define optimal timing or frequency of metabolic monitoring during steroid tapering, emphasizing the need for prospective investigation.

### 8.3. Coordinated Care

Coordinated care between dermatology and endocrinology may warrant consideration in future multidisciplinary models of care for patients with DM who have concomitant diabetes, metabolic syndrome, or steroid-induced hyperglycemia. Whether optimization of glucose management influences cutaneous inflammation, ulcer recurrence, or treatment responsiveness in DM remains unknown and requires prospective evaluation. Interdisciplinary approaches are discussed here as conceptual frameworks rather than evidence-based care pathways.

### 8.4. Patient Counseling and Lifestyle Considerations

Dermatologists play an important role in educating patients about how metabolic factors influence cutaneous disease activity. Future studies may clarify whether patient education regarding metabolic health influences cutaneous disease activity or treatment response in DM. At present, discussions of dietary habits, physical activity, and weight management are best framed as general health considerations rather than disease-specific interventions. Any associations between glucose management, flare intensity, and treatment response in DM remain speculative.

### 8.5. Identification of Worsened Hyperglycemia-Linked Cutaneous Breakdown

Features such as chronic erythema, ulcerations, and impaired wound healing may raise hypotheses regarding the contribution of metabolic disturbances to cutaneous disease persistence. However, these features are not specific to glycemic dysregulation and should not be interpreted as diagnostic indicators. Their relevance to metabolic status in DM remains an area for future investigation.

### 8.6. Summary of Hypothesis-Generating Concepts

The concepts outlined in this section are hypothesis-generating and intended to stimulate future research rather than serve as evidence-based clinical recommendations. Although metabolic screening and glycemic assessment are biologically plausible areas of interest in cutaneous DM, prospective studies are needed to determine their relevance to disease activity, wound healing, and long-term outcomes. Until such data are available, metabolic considerations should be interpreted as investigational rather than practice changing.

## 9. Conclusions

DM is a rare immune-mediated inflammatory myopathy characterized by proximal muscle weakness and distinctive cutaneous manifestations. Its pathogenesis involves autoantibody production, T-cell–mediated inflammation, cytokine dysregulation, and microvascular injury, with emerging evidence supporting a contributory role for metabolic dysfunction.

This review synthesizes evidence supporting hyperglycemia as a determinant of cutaneous DM severity through mechanisms including oxidative stress, advanced glycation end-product accumulation, and amplification of proinflammatory signaling. Together, these processes impair wound healing and exacerbate skin involvement. Although direct interventional data in DM remain limited, existing evidence supports integrating metabolic assessment, glycemic monitoring, and lifestyle-based interventions as adjuncts to dermatologic management to address modifiable risk factors and potentially improve outcomes.

Future research should prioritize longitudinal and interventional studies evaluating glycemic optimization, dietary strategies, and insulin sensitivity in relation to cutaneous disease activity. Such studies should incorporate standardized cutaneous severity measures (e.g., CDASI), clearly defined glycemic phenotypes, and disease-specific populations to better delineate metabolic contributions to DM skin activity. Addressing hyperglycemia as a component of DM pathogenesis may reduce disease burden, optimize management, and improve quality of life. This approach reframes cutaneous severity not solely as an immunologic consequence, but as a multidimensional process shaped by metabolic vulnerability.

## Figures and Tables

**Table 1:** Conceptual links between glycemic dysregulation and cutaneous disease severity in dermatomyositis.

Pathophysiologic Domain	Glycemic/Metabolic Driver	Effect on Skin Biology	Clinical Implication in Cutaneous DM
Inflammatory signaling	Chronic hyperglycemia	Oxidative stress and NF-κB activation leading to sustained cytokine and chemokine production	Increased rash severity and inflammatory flares
Endothelial and microvascular integrity	Advanced glycation end-product (AGE) accumulation and RAGE activation	Endothelial dysfunction, impaired perfusion, and microangiopathy	Vasculopathic changes, ulceration, and delayed lesion resolution
Cellular repair mechanisms	Insulin resistance	Impaired keratinocyte and fibroblast proliferation and migration	Impaired wound healing and reduced response to standard therapies
Immune regulation	Dysglycemia-associated immune imbalance	Persistent pro-inflammatory milieu and altered immune cell function	Chronic cutaneous inflammatory activity and refractory disease
Wound healing and infection defense	Hyperglycemia and metabolic syndrome	Delayed re-epithelialization and increased susceptibility to infection	Chronic ulcerative lesions and secondary skin infection risk
Context of treatment	Corticosteroid-induced hyperglycemia	Secondary metabolic stress compounding inflammatory and vascular injury	Exacerbation of cutaneous disease burden during treatment

**Table 2:** Metabolic and dietary contributors relevant to cutaneous inflammation and disease severity in dermatomyositis.

Factor	Primary Metabolic Effect	Pathophysiologic Mechanism	Potential Impact on Cutaneous DM
Chronic hyperglycemia	Sustained elevation of blood glucose	Oxidative stress, AGE accumulation, RAGE activation, endothelial dysfunction	Increased rash severity, vasculopathic changes, and impaired wound healing
Insulin resistance	Reduced cellular glucose uptake	Chronic low-grade inflammation, impaired keratinocyte and fibroblast function	Delayed re-epithelialization and reduced treatment responsiveness
Metabolic syndrome	Combined dysglycemia, dyslipidemia, central adiposity	Microvascular dysfunction, impaired angiogenesis, inflammatory amplification	Chronic ulceration, poor healing, increased infection risk
High-glycemic index diet	Postprandial glucose and insulin spikes	Increased insulin/IGF-1 signaling, oxidative stress, inflammatory mediator release	Exacerbation of cutaneous inflammatory activity and disease flares
Corticosteroid exposure	Steroid-induced hyperglycemia and insulin resistance	Secondary metabolic stress compounding inflammatory and vascular injury	Worsening cutaneous disease burden during treatment
Obesity	Adipokine imbalance and insulin resistance	Pro-inflammatory adipokine signaling and endothelial dysfunction	Amplified cutaneous inflammatory activity and delayed lesion resolution
Anti-inflammatory dietary patterns (e.g., Mediterranean-style diet)	Improved insulin sensitivity and glycemic control	Reduction in systemic inflammatory markers and oxidative stress	Enhanced skin barrier function and improved healing capacity

## Data Availability

No new data were created or analyzed in this study.
